# Iowa Gambling Task with non-clinical participants: effects of using real + virtual cards and additional trials

**DOI:** 10.3389/fpsyg.2013.00935

**Published:** 2013-12-12

**Authors:** William H. Overman, Allison Pierce

**Affiliations:** Department of Psychology, University of North Carolina WilmingtonWilmington, NC, USA

**Keywords:** Iowa Gambling Task, optimal performance, real cards, virtual cards, non-clinical populations, age, gender, orbital prefrontal cortex

## Abstract

Performance on the Iowa Gambling Task (IGT) in clinical populations can be interpreted only in relation to established baseline performance in normal populations. As in all comparisons of assessment tools, the normal baseline must reflect performance under conditions in which subjects can function at their best levels. In this review, we show that a number of variables enhance IGT performance in non-clinical participants. First, optimal performance is produced by having participants turn over real cards while viewing virtual cards on a computer screen. The use of only virtual cards results in significantly lower performance than the combination of real + virtual cards. Secondly, administration of more than 100 trials also enhances performance. When using the real/virtual card procedure, performance is shown to significantly increase from early adolescence through young adulthood. Under these conditions young (mean age 19 years) and older (mean age 59 years) adults perform equally. Females, as a group, score lower than males because females tend to choose cards from high-frequency-of-gain Deck B. Groups of females with high or low gonadal hormones perform equally. Concurrent tasks, e.g., presentation of aromas, decrease performance in males. Age and gender effects are discussed in terms of a dynamic between testosterone and orbital prefrontal cortex.

## BACKGROUND

### OUTLINE OF PRESENT REVIEW

In this review, we discuss results from over 1,500 non-clinical subjects performing our real/virtual version of the Iowa Gambling Task (IGT), and we compare our results with those from previous IGT studies. First, we describe our laboratory real/virtual card IGT task. The sections of this paper include: (1) a detailed description of our real/virtual card IGT, (2) an experimental comparison of performance on the real/virtual IGT vs. four versions of a commercialized IGT from Psychological Assessment Resources^™^ (PAR^™^ IGT), (3) results from our laboratory using the real/virtual card IGT that study the relationship of participant age and IGT performance, (4) results from our laboratory using the real/virtual IGT of gender differences in performance, (5) a general discussion of this review, and (6) a brief summary.

### DESCRIPTION OF OUR REAL/VIRTUAL CARD IGT VERSION

#### Caveat

The ventromedial prefrontal cortex (VMPFC) and orbital prefrontal cortex (ORBPFC) are not anatomically equivalent (Zald and Rauch, 2006). Nevertheless, in the IGT literature these two areas are often used interchangeably or without anatomical precision. Consequently, in this review we use the designation that is employed in the particular study to which we are referring at that point in the paper.

#### Brief history

Prior to 1997 our laboratory conducted numerous studies that revealed gender differences on cognitive tasks known to be dependent on the integrity of the VMPFC in both children and young monkeys (Overman et al., 1996b, 1997). In order to investigate functions across the life span, we were searching for an adult-level cognitive task that was related to the VMPFC. The IGT was relatively new and especially appealing to our goals because performance was significantly impaired by damage to the VMPFC (Bechara et al., 1994, 1997). In 1997 there was no readily available computerized version of the task so we developed a computerized IGT that followed the exact win–loss sequence used by Bechara et al. (1994, 1997). For more than a year we administered this computerized task to college-aged participants and found little or no learning, i.e., they did not learn to preferentially choose advantageous cards. Rarely did a participant choose more than 60% advantageous cards across 100 trials. When we asked participants about their experience and strategies, they frequently said that they believed the four decks of virtual cards were interactive. For example, they might say “if I choose from Deck A three times in a row, this will change the next card in Deck B and prevent a loss.” The strategies of interactive decks persisted despite our telling participants to treat the virtual decks as real, physical decks of cards. Thus, it appeared to us that with the computerized IGT, subjects based decisions on two things: (1) card value and (2) erroneous strategies of how the decks interacted on the computer.

Obviously, if one were to use real decks of cards, IGT decisions must be based solely on the values of the selected cards (which cannot interact). Consequently, we developed a version of the IGT that employed the simultaneous use of real and virtual cards (real/virtual card IGT). In this task, participants chose from decks of paper cards while an experimenter mimicked their card choice on virtual decks on an adjacent computer screen. The real and virtual cards were prearranged so that each card, when turned over, exactly matched the wins and/or losses as used by Bechara et al. (1994). In addition, the computer kept score of wins and losses and displayed the ongoing total of money.

This technique dramatically improved performance, presumably because it was obvious to the participant that paper decks could not be interactive and, thus, erroneous strategies of deck interaction were eliminated. With the real/virtual card IGT, participants showed significant and progressive learning on the task, gradually increasing their choice of advantageous cards up to approximately 70–80%+ depending on how many trials were administered (Reavis and Overman, 2001; Overman et al., 2004, 2006). Furthermore, we discovered that administration of more than the traditional 100 trials revealed important data not otherwise shown. Not only did overall IGT performance continue to improve, i.e., percentage of advantageous cards continued to increase beyond 100 trials, but clear and significant gender differences in task performance emerged (e.g., Reavis and Overman, 2001). Specifically, females chose significantly fewer advantageous cards from Decks C and D than did males. This gender difference was driven by females’ preference for cards from disadvantageous Deck B, which has a high win-to-loss ratio. Other researchers have confirmed similar gender differences (for review of sex differences on the IGT, see van den Bos et al., 2013).

#### Description of the real/virtual IGT

In our IGT, the subject sits in front of four decks of paper cards, behind which is a computer screen showing four virtual decks of cards. As the subject selects a paper card, the adjacent experimenter selects the same virtual card. The real and virtual cards have exactly the same value of wins and losses. The computer shows a running total of “money.” The real/virtual card IGT version has the identical sequence of wins and losses for every card in the task as used by Bechara et al. (1994, 1997). Each deck contains 40 cards as in Bechara et al. (1997), and a deck can be reused if depleted. Participants start the task with $2,000 in points. There are two advantageous and two disadvantageous decks. Throughout the task, the advantageous decks (Decks C and D) always reward $50 and the disadvantageous decks (Decks A and B) always reward $100. Ten consecutive choices from $50 advantageous Decks C or D result in a net gain of $250; while 10 consecutive choices from $100 disadvantageous Decks A or B result in a net loss of $250. Advantageous $50 Deck D and disadvantageous $100 Deck B contain 10 wins and one loss per 10 trials [analyzed below as “high frequency of gain (HFOG)” decks], while advantageous $50 Deck C and $100 disadvantageous Deck A contain 10 wins and five losses per 10 trials (analyzed below as “low frequency of gain” decks). Consistent selection from $100 disadvantageous Decks A and B results in long-term monetary losses, whereas consistent selection from $50 advantageous Decks C and D result in long-term monetary gains. We use color designation (blue, yellow, green, and red) for the decks (e.g., Reavis and Overman, 2001; Overman et al., 2004, 2011) because pilot studies showed that color names were easier for the experimenter to attend to when mimicking the participant’s choice on the computer. The letter/color variable does not affect performance (Overman et al., 2011, 2013).

## USE OF REAL CARDS AND ADDITIONAL TRIALS INCREASE IGT PERFORMANCE: COMPARISON OF SIX

### IGT VERSIONS INCLUDING THE COMMERCIALLY AVAILABLE PAR^™^ IGT NUMEROUS VERSIONS OF IGT HAVE BEEN USED

The IGT has been used in hundreds of scientific studies but, unfortunately, testing procedures have varied widely. The variations include, but are not limited to, specified details of test procedures, instructions to the participant, number of trials, analysis by gender, analysis of performance in terms of percent advantageous cards vs. a net score, analysis by deck type, the use of real vs. virtual cards, use of real money, and education level of subjects (for reviews, see Fernie and Tunney, 2006; Overman et al., 2013). Perhaps one reason there have been so many IGT versions is that Bechara et al. (1999) did not publish details of procedures, such as instructions, until several years after its introduction, and type of instruction is known to affect IGT performance (see Balodis et al., 2006; Fernie and Tunney, 2006).

### ARE RESULTS EQUIVALENT WHEN USING VIRTUAL AND REAL CARDS

In the original IGT (Bechara et al., 1994), construct assessment was based only on the number of real cards chosen from each deck type. In 2000 a computerized version was utilized (Bechara et al., 2000). Recently, a similar computerized IGT has become commercially available from Psychological Assessment Resources (PAR^™^; 2007). This test is designed as an assessment tool for clinical populations and as a complement to other neuropsychological tests (Bechara, 2007). The PAR^™^ IGT differs from the original IGT on two dimensions: (1) it uses virtual cards and (2) it employs an increasing progression of wins and losses every 10 trials [see Bechara et al. (2000)].

Changes in test instrumentation must proceed with caution. Sometimes such a change can introduce confounding variables (Steinmetz et al., 2010). In the case of the IGT, there may have been unintended consequences of using virtual cards rather than real cards as this has been documented for another well-known test of frontal function, the Wisconsin Card Sorting Task (Steinmetz et al., 2010).

Performance equivalence between test versions is critically important because one of the requirements of a sound assessment tool of a psychological construct is that all versions of the test should use procedures that yield optimal performance for all test takers, i.e., there should be nothing about the *test procedures*, *per se*, that restricts performance. This is a basic element of construct validity. Only by establishing the optimal baseline of decision-making in normal participants can comparisons with clinical populations be accurate. Others have questioned the construct validity of the traditional IGT (e.g., Dunn et al., 2006).

### EXPERIMENTAL EFFECTS OF USING REAL + VIRTUAL CARDS AND EXPLICIT INSTRUCTIONS

There are multiple components of construct validity for the IGT. Among those constructs that have been studied are the definition of decision-making, reliability, and the impact of personality and mood (Buelow and Suhr, 2009). To expand research in this area, we addressed the *validity* component of optimal performance on the IGT, especially the PAR^™^ IGT (Overman et al., 2013). In this study, we compared performance on five versions of the IGT including four versions of the commercially available PAR^™^. Across the five versions, several procedural variables were systematically manipulated: (a) method of delivery (computerized versions vs. versions using real decks of cards); (b) number of trials (100, 200, and 400); (c) instructions given to the participant; and (d) incentives for the subject to perform as well as possible.

This study had two primary experiments. In Experiment 1, we compared performance on five versions of a 100-trial IGT: four versions of the PAR^™^ IGT and one version that more closely resembled the original IGT. In Experiment 2, we compared performance on the 100-trial IGT used in our laboratory with performance on the same IGT with 200 trials and an additional 200 trials plus incentive. In the first experiment, 214 male and 364 female college students were randomly assigned to one of five versions of the IGT. Full descriptions are presented in Overman et al. (2013), but a brief description is necessary for understanding of our data:

***IGT Version 1.*** Commercially available computerized PAR^™^ version of the 100-trial IGT using the standard instructions included with the PAR^™^ IGT: the participant was told that some decks are “worse than others” and that they were “to try to win as much money as possible and avoid losing money as much as possible.”

***IGT Version 2.*** PAR^™^ IGT (100 trials) With Explicit Instructions: the participant was told there were two types of decks, “good” and “bad” and that if they consistently chose from the good decks, they would win more money than they would lose and that their goal was to figure out which were good and bad decks to win as much money as possible.

***IGT Version 3.*** PAR^™^ IGT (100 trials) with original PAR^™^ instructions, but using paper cards from four tangible decks.

***IGT Version 4.*** PAR^™^ IGT (100 trials) with explicit instructions *and* with paper cards from four tangible decks.

***IGT Version 5.*** IGT traditionally used in our lab using paper cards and virtual cards (real/virtual card IGT; 100 trials). Version 5 employed an identical pattern of wins and losses across all cards as in the original version of the IGT in that Decks A and B always paid $100 and Decks C and D always paid $50 (Bechara et al., 1994). In addition, if a deck was depleted, it could be reused, giving the participant a choice between all four decks. The instructions were identical to those used in IGT Version 2.

[Note: For all of the PAR^™^ versions, each deck contained 60 cards and if a deck was depleted, the participant was forced to choose from the remaining three decks. In addition, the PAR^™^ decks paid an *average* of $100 or $50; the net loss or gain from each deck increased across each block of 10 cards. For example, for Deck A, at the outset of the test, the total gain for Block 1 is $1000 and the total loss for Block 1 is $1250 for a net loss of $250. In each subsequent block of 10 trials the average gain increases by $10 per block, i.e., the average win in Block 2 will be $110 and in Block 3 the average win will be $120 and so forth. In addition beyond the first block, the number of losses increases by one card per block. Thus, there are six losses in Block 2, seven losses in Block 3 and so on. The total net loss per block increases by $150 such that for Block 2 the net loss is $400, rather than the net loss of $250 in Block 1 and for Block 3 the net loss is $550 and so on. While the number of losses increases from block to block, the amount of the loss per card remains within the range of $150–350 for each block. The incremental changes in gains and losses continue through all six blocks so that the total net loss for 60 cards in Deck A is $3750. The progression of wins and losses is explained for each deck in the PAR^™^ IGT manual (Bechara, 2007).]

Note: The PAR^™^ IGT has optional “slot machine” sounds that can accompany the visual display of wins and losses; however, these sounds were not employed in any version of the task in our study.

### RESULT #1: NO EFFECT OF TYPE OF INSTRUCTION

A 2 (gender) × 5 (IGT Version) × 4 (blocks of trials) was conducted. As discussed below, there was a significant effect of (a) IGT Version, (b) blocks of trials, (c) an interaction between gender and block, and (d) an interaction between version and block.

There was no significant effect of the nature of instructions, explicit or not. This was shown by the dual facts that (1) performance was not statistically different on Version 1 (PAR^™^ IGT) and Version 2 (PAR^™^ IGT + explicit instructions), and (2) performance was not statistically different on Version 3 (PAR^™^ IGT with cards and regular instructions) and Version 4 (PAR^™^ IGT with real cards and explicit instructions).

It is important to note that all instruction types employed “hints” about what the subject was expected to do. Fernie and Tunney (2006) reported that IGT instructions including a hint about the nature of the task significantly improved performance relative to instructions with no hint. The hint referred to by Fernie and Tunney (2006) concerned instructing the subject that “some decks are worse than others and you can win if you stay away from the worst decks.” In the present study both types of instructions contained a similar hint. The PAR^™^ IGT instructions were essentially the same as those used by Bechara et al. (1999) and said “the goal of the task is to win as much as possible and lose as little as possible; some decks are worse than others; you will win if you stay away from the worse decks.” The “explicit” instructions we used in IGT Versions 2, 4, and 5 said “there are good decks and bad decks; if you pick from the good decks you will win more money than you lose, but if you pick from the bad decks you will lose more money than you win; your job is to figure out which are the good decks and which are the bad decks.”

It is possible that hints may affect the degree of awareness (Dunn et al., 2006; Persaud et al., 2007), which in turn, could affect performance. Perhaps the inclusion of similar “hints” in the two instructional sets in this study eliminated any performance effect of this variable. Nevertheless, in our comparison of IGT versions, there was no systematic difference in IGT performance between versions that used originally published instructions (Bechara et al., 1999, and PAR^™^ IGT) or “explicit” instructions.

### RESULT #2: USE OF REAL CARDS + VIRTUAL CARDS ENHANCES PERFORMANCE

**FIGURE 1:**
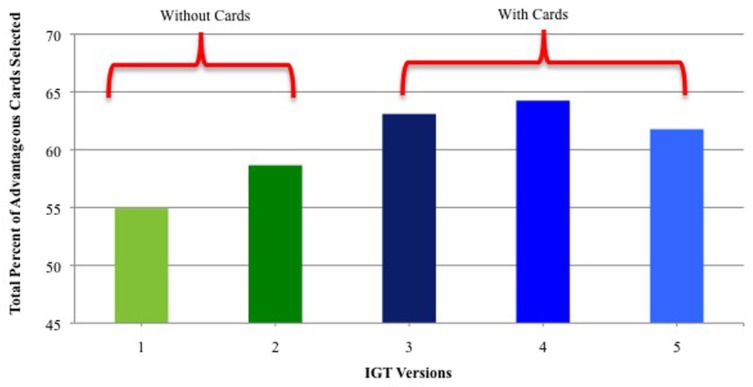
**Percent of advantageous card selections for IGT versions that used only virtual cards (Versions 1 and 2) and versions that used real + virtual cards (Versions 3 –5).** Vertical bars indicate standard error of the mean (SEM).

IGT performance (percent of advantageous cards selected: Decks C + D) was significantly higher when real/virtual cards were used vs. virtual cards alone. As shown in **Figure 1**, performance was higher in IGT Versions 3, 4, and 5 than in Versions 1 and 2 (when only virtual cards were used). There were no significant performance differences on Versions 1 and 2.

### RESULT #3: USE OF REAL/VIRTUAL CARDS PROMOTES LEARNING THROUGHOUT THE 100 TRIAL TASK

**FIGURE 2:**
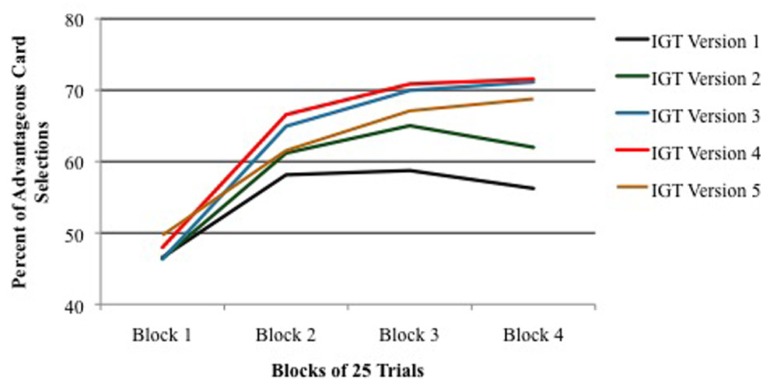
**Percent of advantageous card selections across four blocks of 25 IGT trials for each version of the task.** Vertical bars indicate SEM.

**FIGURE 3:**
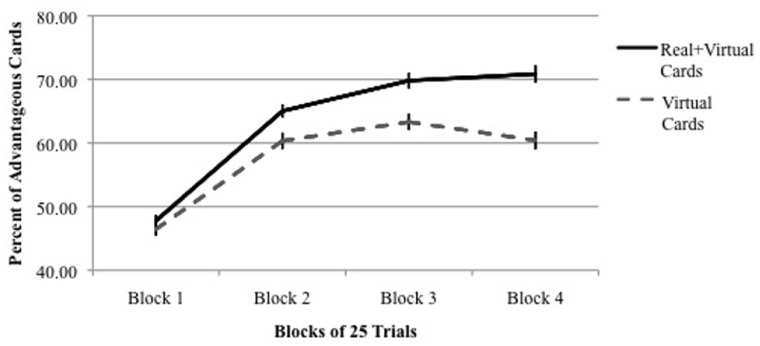
**Percent of advantageous card selections across four blocks of 25 IGT trials using real + virtual cards vs.** virtual cards only. Vertical bars indicate SEM.

Another finding emerged from the analysis of performance across four blocks of 25 trials each. As shown in **Figure 2**, performance was equal among all IGT versions during the first block of 25 trials (the exploration period), but in all versions, performance in Block 2 was significantly higher than performance in Block 1. In other words, in each IGT version learning occurred within the first 50 trials. However, as shown in **Figure 2**, when real cards were used (Versions 3–5), learning continued to improve in Blocks 3 and 4. In contrast, when only virtual cards were used (Versions 1 and 2), performance leveled off for the remainder of the trials after the second block. This result is more dramatic when presented as a comparison between combined versions using real/virtual cards (Versions 3–5) and combined versions using only virtual cards (Versions 1 + 2; **Figure 3**).

One important feature of our real/virtual card IGT should be emphasized at this point. In our task, if a deck was depleted, it was turned over and could be reused. This is not the case for the PAR^™^ IGT in which a depleted deck cannot be reused. This means that the participant would then be forced to choose between three decks, some of which might not be his/her preferred advantageous deck type. This situation would penalize subjects that learn early in the game as noted by Dunn et al. (2006).

### WIDESPREAD ASSUMPTION OF EQUIVALENT RESULTS WITH REAL AND VIRTUAL CARDS

The data presented above raise an important point that is relevant for all research using the IGT. Until now, there has been a widespread assumption in the IGT literature that performance is equal when using real or virtual cards. Two specific IGT papers have been frequently cited as the basis for this assumption: Bowman et al. (2005) and Bechara et al. (2000).

#### Bowman et al. (2005)

Bowman et al. (2005) compared IGT performance with real cards and with a computerized format. They reported no significant difference in performance between the two formats. However, these results are difficult to interpret because of the low number of subjects and the almost exclusive use of females. There were only 22 subjects in each of three experiments. Across all experiments there were 56 females and 10 males, i.e., 85% female. Given the consistent finding that females do not perform as well as males on the IGT (Reavis and Overman, 2001; Bolla et al., 2004; Overman, 2004; Overman et al., 2004, 2006), the data from Bowman et al. (2005) may represent something of a floor effect among groups. This lower compression of scores may have obscured significant differences between groups that might have been apparent if there had been more subjects and a balanced number of males and females.

#### Bechara et al. (2000)

**FIGURE 4:**
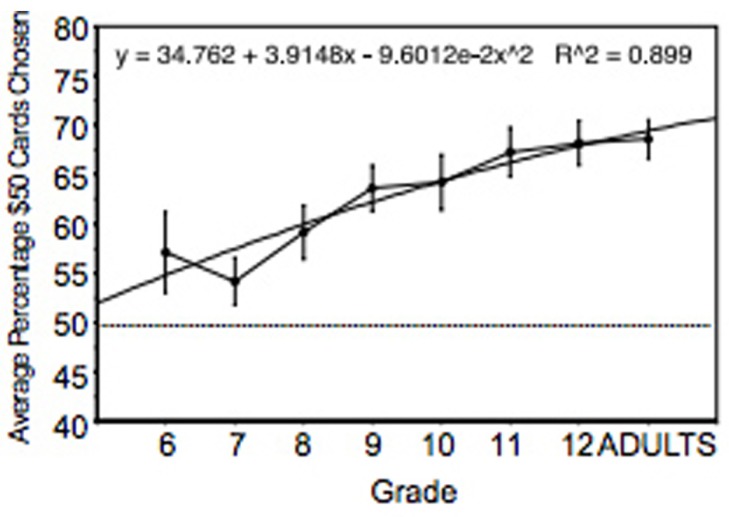
**Percent of advantageous card selections across adolescence and young adulthood.** The regression equation shows that age accounted for 90% of the variance. Vertical bars indicate SEM.

Published papers frequently cite Bechara et al. (2000) when stating that IGT performance is equal when real or virtual cards are used (Bechara, 2007; Schneider et al., 2007; Buelow and Suhr, 2009). Close analysis of the paper by Bechara et al. (2000) shows a different picture. The authors tested normal participants and patients with damage to the VMPFC on two IGT versions using real cards versions [A, B, C, D and E, F, G, H (card task with different order of cards and payments from original A, B, C, D)] and two computerized IGT versions using virtual cards versions [A’B’C’D’ and E’F’G’H’]. Not only were the latter two versions computerized, they also introduced progressive wins and losses for every 10 trials. In other words, two factors were changed during the switch to a computerized task: (a) real vs. virtual cards and (b) stable vs. increasing wins and losses per block. As expected, in all four versions of the IGT, VMPFC patients were impaired relative to normal subjects. In this regard, the authors write: “the results from the computer tasks mirrored those from the original task (ABCD) and variant (EFGH) task” (Bechara et al., 2000, p. 2197). Given the context of the paragraph, the authors appear to be documenting the fact that VMPFC patients were impaired relative to normal participants regardless of whether real vs. virtual cards were used or whether wins and losses were stable or progressive. However, this does not mean that normal participants performed equally well with real vs. virtual cards. In fact, their data indicate that normal participants performed better when real cards were used. As shown in Figure 4A (Bechara et al., 2000, p. 2197), normal participants’ learning leveled off after the first two blocks of trials when virtual cards were used; however, with real cards, normal participants’ learning increased throughout the task from Block 1 to 5 Figure 2A, p. 2195). The paper contained no statistical analyses of the data. However, inspection of the SEM bars indicates two important things: first, when real cards were used, there was little or no overlap, block to block, from Block 1 through 5 Figure 2A, i.e., learning continued after the second block and throughout the task. Secondly, when virtual cards were used, there was considerable overlap in Blocks 2–5 Figure 4A, i.e., learning plateaued after the second block. So, it appears that normal participants performed better when real cards were used than when virtual cards were used.

***Caveat.*** The virtual card version employed progressive wins and losses which the real card versions did not. Although the progressive win/loss schedule in Bechara et al. (2000) was not described, it may have been similar to the progressive version of the PAR^™^. So the differences in performance in normal subjects may have been due to either the real/virtual variable or the progressive consequences variable. Our study only compares the variable of real vs. virtual cards and we show that the card variable is critically important for IGT performance.

### EFFECTS OF ADMINISTERING MORE THAN 100 TRIALS ENHANCES IGT PERFORMANCE

In part A of the second experiment by Overman et al. (2013), the same subjects who participated in 100 trials of the Version 5 IGT were given an additional 100 trials. There was a significant effect for both number of trials and gender (discussed in Section “Gender Differences on IGT Performance: Deck-by-Deck Analysis”). In the first set of 100 trials, participants chose an average of 62% advantageous cards. This significantly increased to 72%. Furthermore, in the last (eight) block of trials, males and females chose 85 and 67% advantageous cards, respectively. These results clearly show that IGT performance is significantly enhanced with the addition of extra trials. In addition, males outscored females in the last block of 100 trials, and they continued to do so throughout the second set of 100 trials. This indicates that females did not “catch up” with the males even given additional trials, i.e., the female difference was not simply due to a slow start in performance. Most importantly, the continued increase in selection of advantageous cards during the second 100 trials by all subjects means that the decision-making process was not complete after only 100 trials. If the purpose of the IGT is to “measure decision-making,” one presumes it is meant to assess *complete* or *finished* decision processes. Our results indicate that for the IGT, decision-making processes are not complete until well after 100 trials. Others have noted that the administration of more than 100 trials might reveal important insights of different populations, e.g., that patient groups may be slow to learn and show increased performance beyond 100 trials (Dunn et al., 2006).

### SUMMARY OF SECTION “USE OF REAL CARDS AND ADDITIONAL TRIALS INCREASE IGT PERFORMANCE: COMPARISON OF SIX”

Iowa Gambling Task performance is maximized when real/virtual cards are used and there are more than 100 trials. This real/virtual card procedure is inconvenient as compared to a simple computerized IGT, in part because the task requires an experimenter to mimic responses on the computer. However, convenience is not a substitution for complete and accurate assessment of performance.

## EFFECTS OF AGE ON THE IGT

### EXPERIMENTAL EXAMINATION OF PERFORMANCE ON REAL/VIRTUAL CARD IGT FROM EARLY ADOLESCENCE THROUGH OLD AGE

Because of its sensitivity to decision-making impairments among patients with circumscribed brain damage, the IGT has been used as a behavioral proxy for brain development across the life span (Wahlstrom et al., 2010). The traditional IGT is too complex for young children, so simplified versions have been developed for this population. Traditional IGTs, including our real/virtual card version, have been administered to participants from early adolescence to old age.

### PERFORMANCE OF CHILDREN ON VARIATIONS OF THE IGT

To our knowledge, the real/virtual card IGT has not been administered to children. But it is important to review, if even briefly, findings from children who perform age-appropriate, “child-friendly” versions of the IGT. These studies consistently show increases in performance within several age ranges: from ages 3 to 4 years (Kerr and Zelazo, 2004), from ages 3 to 5 years (Hongwanishkul et al., 2005), and from ages 6 years to adulthood (Crone and van der Molen, 2004). The latter study employed a widely used child IGT version known as the “hungry donkey task.” In this task, subjects choose between four virtual doors that reveal wins and losses of apples to feed a hungry donkey. The win/loss schedule is essentially the same as in the traditional IGT. On this task, adults (ages 18–25) performed significantly better than adolescents (ages 13–15), who performed significantly better than both an older group of children (ages 10–12) and a younger group of children (ages 6–9). Younger and older groups of children performed equivalently (Crone and van der Molen, 2004). In a study by Garon and Moore (2004), 3-, 4-, and 6-year-old children were given a 40-trial variant IGT that involved “bears” and “tigers” and candy rewards. There were no significant age or gender differences in performance. There was a block × gender effect in which females made more advantageous choices than males in the second block of 20 trials. The meaning of this finding is unclear. The authors acknowledge that their task was quite different from the IGT in terms of the nature of instructions and performance feedback and those differences may have contributed to the unexpected gender effect.

### PERFORMANCE ON REAL/VIRTUAL CARD IGT FROM ADOLESCENCE TO OLD AGE

Since there are substantial brain changes, especially in the prefrontal cortex (PFC), during adolescence, the IGT is an ideal task to use with this population. Indeed, several hypotheses attribute poor decision-making among adolescents to neuroanatomical changes in areas within the PFC. Optimal performance on the IGT is dependent on the integrity of several regions in the PFC including the ORBPFC (Bechara et al., 1994), the dorsolateral (DL) PFC (Fellows and Farah, 2005), or dorsomedial (DM) PFC (Manes et al., 2002). Damage to any of these areas impairs IGT performance as defined by selection of more cards from disadvantageous decks than from the advantageous decks. These brain areas and others change during adolescence. In general, during this period, cortical gray matter increases and decreases at somewhat different schedules in different brain regions (Giedd et al., 1999). In the frontal cortex, overall gray matter increases during early adolescence and peaks at age 12 for males and age 11 for females (Giedd et al., 1999). This peak is followed by a decrease in cortical gray matter volume until late adolescence.

However, the specifics of prefrontal development are exceedingly complex. The frontal cortex is heterogeneous and not all sub-areas develop simultaneously during adolescence. There is regionally specific development with some areas being pruned, while other areas are showing increases in synapses (Giedd et al., 1999). Some researchers have suggested that changes in DLPFC are most highly correlated with adolescent behavior patterns (e.g., Lewis, 1997; Sowell et al., 2001; Paus, 2005) while others have suggested that changes in VMPFC are most highly correlated to such patterns (Hooper et al., 2004; Schwartz et al., 2010). Our research with IGT performance during adolescence was not designed to determine the underlying neural bases for behavior changes. Rather we have studied behavioral changes in IGT performance throughout adolescence.

In two studies, detailed below, we administered the real/virtual card IGT (with more than 100 trials) to non-clinical participants ranging in age from 11 to 62 years. In the first study, we administered 200 trials of the real/virtual card IGT to children in the sixth through the 12th grade, as well as to college students (Overman et al., 2004). In the second study, we administered 150 trials of the real/virtual card IGT to adults ranging from college-age to 60+ years (Reavis and Overman, 2001). In that study, hormone levels were determined from blood samples for young women (low or high hormone) and older women (with or without estrogen replacement therapy, ERT).

### AGES 11–23 YEARS: PERFORMANCE OF ADOLESCENTS ON REAL/VIRTUAL CARD IGT

We measured the performance of adolescents in sixth through 12th grade (11–18 years) and college students (17–23 years) on 200 trials of our real/virtual IGT and a control task, the Wisconsin Card Sorting Task (WCST; Overman et al., 2004). In addition, we administered surveys of impulsivity and excitement-seeking (impulsivity and excitement-seeking subscales of the NEO Personality Inventory; Costa and McCrae, 1992). The WCST was used as a control task for generalized executive dysfunction because it is more dependent upon DLPFC systems than on VMPFC systems (Cabeza and Nyberg, 2000, but see Manes et al., 2002; Fellows and Farah, 2005 for evidence of IGT impairment following damage to the DLPFC). Thus, normal performance on the WCST plus impaired performance on the IGT would suggest localized dysfunction (VMPFC) rather than a generalized prefrontal dysfunction. A number of studies have reported little relationship between IGT and WCST performance measures (see Bechara, 2007), although there may be some correlation between WCST and performance in the last blocks of the IGT (Brand et al., 2007).

Performance was analyzed using the percentage of advantageous cards (C + D) selected across 200 trials. As shown in **Figure 4**, there was a steady and statistically significant increase in performance across age. Performance of sixth and seventh grade participants was significantly lower than performance of participants in the ninth grade and above. In addition, performance of participants in the eighth grade was significantly lower than participants in the 11th grade and higher. Performance of participants in the ninth grade and higher were equivalent. There were 30 males and 30 females in each age group and we speculate that the use of more subjects would have revealed additional statistical differences between groups.

Furthermore, an analysis of performance in each block of 50 trials showed, across subjects, significantly lower performance in Block 1 as compared to Blocks 2–4; performance in Blocks 2 and 3 were statistically equivalent, but significantly lower than that in the last block of trial. In other words, as shown above, performance improved throughout the 200 trials on the real/virtual card IGT.

In addition to finding significant effects of age on IGT performance, a gender difference also emerged. These will be discussed more thoroughly in the following section. We did not find a significant correlation between performance on the IGT and measures of substance use, impulsivity, or excitement-seeking. This lack of a significant correlation was due to two factors: (a) there was relatively little substance use within this particular cohort who volunteered to stay after school hours to be tested and (b) because 25 pair-wise correlations were run and thus, the risk of a type 1 error was substantial, the standard correction for multiple correlations generated a stringent criterion alpha level of 0.0009. In other studies there is evidence that IGT performance is negatively correlated with substance use (e.g., Bechara and Martin, 2004) and impulsivity (e.g., Best et al., 2002). In addition, we did not find a significant correlation between IGT and WCST performance.

The steady increase in IGT performance throughout adolescence to young adulthood can be interpreted in many ways, one of which is that increases in performance are related to the ongoing neuroanatomical and neurochemical development of the frontal lobe. Regardless of the interpretation, the data unambiguously reveal a clear distinction between adolescent and young adult participants.

Our findings with adolescents have been replicated (Hooper et al., 2004). In that study, participants were given 100 trials (five blocks of 20 trials) of a computerized IGT with a contingency value scaled below the traditional IGT in order to employ real monetary rewards or punishments. Overall, 14- to 17-year-old participants performed significantly higher than 9- to 10-year-old participants. In Block 4, the 14- to 17-year-old group performed better than both the 9- to 10- and 11- to 13-year-old groups. In Block 5, the 14- to 17-year-old group performed better than only the 9- to 10-year-old group. These data confirm our finding that older adolescents learned the task earlier and to a greater extent than younger participants.

### AGES 19–63 YEARS: PERFORMANCE ON REAL/VIRTUAL CARD IGT FROM EARLY ADULTHOOD TO OLD AGE

In a separate study of possible age-related changes in IGT performance we tested non-clinical adults ranging in age from 19 to 63 years (Reavis and Overman, 2001). These subjects were also tested on a probability-learning task, the California Weather Task (CWT; Knowlton et al., 1994, 1996a,b). The WT was chosen for two reasons: first, as is the case for the IGT, learning is gradual across multiple trials. Secondly, as is the case for the early trials of the IGT, individuals can learn without being aware of the information they have acquired. This notion is an essential component of the somatic marker hypothesis (SMH; Damasio et al., 1991). In contrast to the IGT, performance on the WT is dependent upon the dorsal striatum (Packard et al., 1989), and, as such, is impaired in Parkinson’s and Huntington’s patients, but not in amnestic adults (Knowlton et al., 1994). The CWT is a probabilistic classification habit task. Participants are shown up to four cards on a computer screen and must gradually learn which combinations of cards predict one of two weather outcomes: rain or sunshine. A particular card is associated with the outcome of sunshine 75, 57, 43, or 25% of the time, and thus, associated with the outcome of rain 25, 43, 57, and 75% of the time. Participants are exposed to any combination of cards on a given trial, and they must gradually learn which cards and combinations are probabilistically related to a given outcome. The computer provides visual and auditory feedback corresponding to a correct or incorrect response.

In addition to the real/virtual card IGT and WT, sensation-seeking (sensation-seeking scales; Zuckerman, 1979), and depression (Center for Epidemiological Studies Depression Scale; Radloff, 1977) were assessed as well as hormone status (estradiol, progesterone, and testosterone were assayed from blood samples). This resulted in six distinct groups as described in the Section “Gender.” The groups were: (1) young males, mean age 19.1 years; (2) older males, mean age 59.4 years; (3) young menstruating females, mean age 19.8 years; (4) young mid-luteal females, mean age 22.4 years; (5) older women on ERT, mean age 54.5; (6) older women not on ERT, mean age 62.7. The order of the IGT and WT were counterbalanced. Performance was measured by the percentage of advantageous cards (from Decks C + D) across 150 trials. Additionally, rule-stating was measured by asking the participant to tell the experimenter “all they knew about the game and how they felt about the game” at intervals of 10 trials. If they did not state which two decks they thought were good or bad, they were prompted to do so. After the response they were reminded that the good and bad decks always remained the same. We recorded at what point during the task the rule was stated correctly, i.e., that Decks C and D were the “advantageous”, or “best”, etc., decks.

**FIGURE 5:**
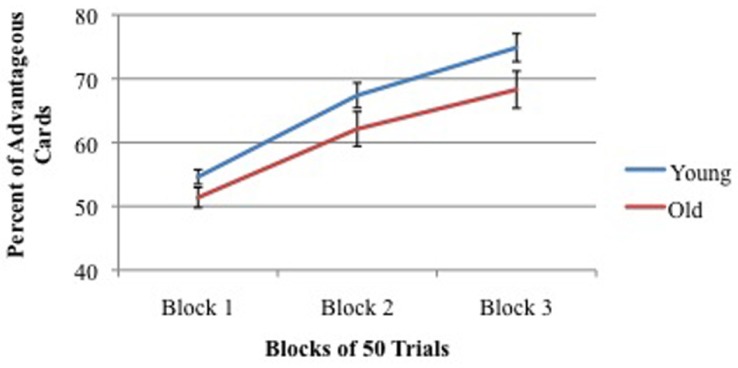
**Percent of advantageous card selections across three blocks of 50 trials for young (mean = 19 years) and old (mean = 59 years) participants.** Vertical bars indicate SEM.

There was no significant effect of age on IGT performance between young adults (groups 1 + 3 + 4) and older adults (groups 2 + 5 + 6). Nor was there a significant effect of hormones within groups of males or females. Across 150 trials, young participants selected 65.6% advantageous cards and older participants selected 60.6% advantageous cards (Reavis and Overman, 2001). As shown in **Figure 5**, both young and old participants improved performance across blocks of trials.

There were no differences in IGT performance among the four hormonal groups of women, so all were collapsed into one group. Similarly, there was no significant difference IGT performance between the two groups of men so both were collapsed into one group. A comparison of males vs. females revealed a significant gender difference. This is discussed in detail below, but essentially men and women were equal in performance on the first block of performance but males performed significantly better than females on the second and third blocks.

While there were no significant age differences in adults in IGT performance, there was a significant age difference in rule stating. Significantly more college-age participants (*M* = 63%) stated the correct rule at some point throughout the task than did older participants (*M* = 38%), indicating perhaps a higher level of cognitive awareness of the task and supporting the claim by Dunn et al. (2006) that the IGT is not cognitively impenetrable. In addition, there was an interesting test order effect as well, but only for men. When the CWT preceded the IGT, younger men improved in their IGT performance. This is in contrast to older men’s IGT performance, which declined when the CWT preceded the IGT (Reavis and Overman, 2001). Since fatigue does not seem to be a viable explanation, it appears that having the CWT first was somehow a benefit to younger men and a detriment to older men. At this time, we do not have an explanation for the order effect, and it is an important topic for future research because it indicates that administration of multiple tests might affect performance on the IGT.

With regard to the questionnaires, we found that (1) across all subjects, sensation-seeking was significantly correlated with performance on the real/virtual IGT (*r* = 0.162, *p* = 0.025), (2) males scored higher than females on this scale, and (3) depression scale scores were not correlated with performance on either the IGT or WT (Reavis and Overman, 2001).

#### Comparison with previous studies of young vs. older adults

There are mixed reports about the performance of young and older adults on the IGT.

***Failure to document age changes on IGT.*** Some studies have been consistent with our finding of no age differences. For example, when using the computerized PAR^™^ IGT, Wood et al. (2005) found no performance differences between young adults (ages 18–25) and older adults (ages 65–88). There have been some indications for age changes in tasks that rely on DLPFC in the face of no age changes for tasks that rely on VMPFC. MacPherson et al. (2002) tested young (mean age = 28.8 years), middle-age (mean age = 50.3 years) and older (mean age = 69.9 years) adults on two batteries of frontal tasks: (1) “DLPFC tasks”: WCST, Self Ordering Pointing Task, and Delayed Response and (2) so called “VMPCF tasks”: IGT (using real cards), Faux Pas Task, i.e., detecting social slips, and an Emotional Identification Task. The results revealed age-related declined on all three DLPFC tasks but no age-related changes on the VMPFC tasks with the exception of identifying sadness on the Emotional Identification Task (MacPherson et al., 2002). A somewhat similar study found partially contrasting results. Lamar and Resnick (2004) tested young (mean age = 28 years) and older adults (mean age = 69 years) on four DLPFC tasks and three orbitofrontal (OFC) tasks. There were no age differences on any of the DLPFC tasks but there were some age differences on the OFC tasks. Specifically younger adults scored better than older adults on delayed matching and delayed non-matching to sample tasks. However, there were no age differences on the OFC task of the IGT. In fact, both young and older adults performed exactly the same on the IGT, and chose 55% advantageous cards (Lamar and Resnick, 2004). The selection of only 55% advantageous cards across 100 trials seems to be low compared to most other IGT findings. The authors mention “decks of cards” but it is not clear whether this referred to real paper cards or virtual cards. Finally, Kovalchik et al. (2005) found no performance differences between young adults (18–26 years) and elderly adults (70–95 years) when using a two-deck variation of the IGT.

***Documentation of age changes on IGT.*** In contrast to the failure to find age-related IGT changes, there are a few reports of IGT impairments among some older adults, at least when defined by subgroups of older adults. Denburg et al. (2006) found that there were two significantly different groups among older adults: impaired (as indicated by a negative net score) or unimpaired (as indicated by a positive net score). Similarly, Denburg et al. (2005) found that a subset of older adults (56–85 years of age) showed impairments on the IGT relative to younger adults (26–55 years of age). Specifically, they found that 14 out of 40 (35%) of the adult group were impaired while the majority (65%) was unimpaired. These results were supported by Fein et al. (2007), who found a greater number of adults between the ages of 56 and 85 years were impaired on the IGT in comparison to adults between the ages of 18–55 years. Thus, some, but not all, older adults are reported to show IGT impairments. However, the same can be said of young healthy adults. Some, but not all, young adults score poorly on the IGT (in terms choice of advantageous card selection) and prefer decks with infrequent losses also, as documented by Steingroever et al. (2013) and by Reavis and Overman (2001).

### SUMMARY OF SECTION “EFFECTS OF AGE ON THE IGT”

Age clearly impacts IGT performance, as shown by the differential levels of performance of adolescents through young adulthood (Hooper et al., 2004; Overman et al., 2004). However, with regard to older adults, there are mixed results depending on procedure and type of analysis. In the brief review cited above, four studies failed to find age differences on the IGT and three found age differences in subgroups of older adults. When using our real/virtual IGT, we find little or no evidence for age-related IGT decrements beyond young adulthood.

## GENDER DIFFERENCES ON IGT PERFORMANCE: DECK-BY-DECK ANALYSIS

### BASIC FINDINGS

Both normal males and females show learning on the IGT across 100 or 200 trials, in that they learn to select significantly more advantageous cards than disadvantageous cards. However, males perform at higher levels than females. Gender differences in performance on the IGT were first documented by Reavis and Overman (2001) and have been replicated frequently (for review, see van den Bos et al., 2013). While males, as a group, choose significantly more advantageous cards than do females, there is always overlap on IGT scores for populations of males and females (e.g., Reavis and Overman, 2001; van den Bos et al., 2013). The male bias had been documented with real/virtual cards (e.g., Reavis and Overman, 2001; Overman et al., 2004, 2006) or virtual cards (e.g., Bolla et al., 2004; Denburg et al., 2009).

In terms of card selection, the sex difference is the result of females’ preference for HFOG cards, either from disadvantageous Deck B (van den Bos et al., 2013) or from both HFOG decks, disadvantageous Deck B + advantageous Deck D (Reavis and Overman, 2001; Overman et al., 2006). In terms of a biological basis for these performance differences, there are a number of hypotheses that are discussed below.

The general IGT literature is muddled with reference to gender differences for several reasons. First, many studies have not analyzed IGT performance for gender (Bechara et al., 1997, 2000; Nagy et al., 2006). Secondly, some studies have analyzed for gender and failed to find a difference; however, there was no deck-by-deck analysis (van Honk et al., 2003). Additionally, some studies have conducted a deck-by-deck analysis but not a gender analysis (Wilder et al., 1998; Lin et al., 2007). In the article by Lin et al. (2007), the lack of gender analysis is of considerable concern, because the authors make a particular note that HFOG *disadvantageous* Deck B is selected more than, say *advantageous* Deck C, and results in a “prominent Deck B phenomenon.” Unfortunately, there is no way of knowing whether the preference for HFOG decks was driven by females or not. As discussed below, IGT analyses by gender, deck type, and blocks of trials are essential for the formulation of refined hypotheses about how and why various groups display differential performance.

### IGT GENDER DIFFERENCES ACROSS THE LIFE SPAN

There is evidence that the sex difference in IGT performance persists from childhood to young adulthood, and perhaps longer. Males as young as 7–15 years of age years perform significantly higher than females on a child-friendly variant of the IGT, the hungry donkey task (Crone et al., 2005). Adolescent males (11–18 years of age; Reavis and Overman, 2001) as well as adult males (18–62 years of age) choose significantly more advantageous cards than do females on a 200 trial real/virtual card IGT.

### INTERPRETATION OF FEMALE PREFERENCE FOR HFOG DECKS

At this point in time, it is not known precisely why females as a group tend to prefer HFOG cards relative to males. However, several possibilities can be ruled out: gender differences in math ability, response perseveration, and hormones.

#### IGT gender differences are not due to differential math ability

The IGT can be classified as employing “arithmetic” cards because every card contains a “plus” value and many have a “minus” value. Thus, the participant must have rudimentary calculation skills to determine which decks are “paying off.” Several studies have shown a male advantage in certain mathematical domains (Hedges and Nowell, 1995; Benbow et al., 2000). So, perhaps males make these calculations more rapidly and accurately than do females and, thus, are more efficient on the IGT. To test this theory, we created a new real/virtual card IGT version in which every card contained a win and a loss, and thus required a calculation (Overman et al., 2006). The net outcome of each card and deck matched the corresponding card and deck in the original IGT (Bechara et al., 1994). Since females were inferior to males on the traditional IGT (that requires calculation on 30% of the cards), then one would predict that they would score even more poorly on the new version when calculation was required on 100% of the cards, if females’ poorer performance were due to differential math abilities. Two hundred trials of this real/virtual card IGT were administered to 31 females and 30 males ranging in age from 17 to 29 years. Results showed that across 200 trials, both males and females learned the task; females did not perform significantly differently than men in terms of choosing advantageous cards, although the trend approached significance, *p* = 0.08, with males selecting 69% advantageous cards with females selecting 62% advantageous cards, which is similar to results on the normal IGT. A finer analysis of card choices revealed the gender difference previously found in our laboratory. Specifically, females chose significantly more cards from disadvantageous Deck B than did males. Moreover the magnitude of this difference increased as the task progressed so that in the last block of trials females chose almost twice as many cards from Deck B than did males (25 vs. 13%). Thus, additional math requirements did not aid nor hinder females’ IGT performance relative to that of females with traditional math requirements.

#### IGT gender differences are not due to differential response perseveration

Infant female non-human primates (Clark and Goldman-Rakic, 1989) and infant female humans (Overman et al., 1996b) perseverate significantly more than respective males on reversal tasks that rely on the ORBPFC. This phenomenon may be relevant for adult females’ differential preference for HFOG disadvantageous cards in Deck B. In the original IGT (Bechara et al., 1994), within the first 10 trials, the $1250 penalty card is the ninth card in Deck B. Because almost all participants explore all decks in the first 20–40 trials, the first penalty card from Deck B is typically not selected until well into the task, e.g., on average the 36th draw (Bechara et al., 1997) and the 26th and 29th draw for males and females (Overman et al., 2006). Until these relatively late trials, cards in Deck B have always paid $100, while cards in the other decks have been both rewarded and punished. Thus, participants may gain a sense that Deck B has great positive weight, which may lead to perseveration on this card throughout the task. This may be more likely in females than in males. In other words, they may not reverse preferences as early as men, as is the case for monkeys and infants in object reversal tasks cited above. Several authors have claimed that impaired IGT performance of patients with VMPFC damage is the result of a deficit in reversal learning (Rolls, 1999, 2005; Fellows and Farah, 2005).

This hypothesis has been directly tested with brain-damaged patients. Fellows and Farah (2005) administered the IGT to subjects with VMPFC damage. These authors hypothesized that these patients perform poorly on the IGT because (a) they do encounter high paying cards from Deck B several times without a penalty in the early trials, and (b) they have deficits in shifting their choices to low paying advantageous cards. Indeed, when penalty cards were moved to the front of the decks in the IGT, the VMPFC patients performed as well as normal controls (Fellows and Farah, 2005).

We tested this perseveration hypothesis in normal participants, by creating a new version of the real/virtual card IGT in which the $1250 loss card from Deck B was moved to the third place in the first block of 10 trials and to the first place in Blocks 2–4 (Overman et al., 2006). College-aged males (*n* = 31) and females (*n* = 30) were given this task. Results showed that the position manipulation did not alter sex differences. Although both males and females learned the task, i.e., choose increasingly more advantageous cards as the task progressed, females still chose significantly more cards from Deck B than did males, which resulted in a significantly lower overall performance than males. Thus, differential response perseveration does not appear to be the reason for IGT gender differences.

#### IGT gender differences are not due to hormones within gender

Although there are consistent findings of sex differences on the IGT, an analysis of high and low gonadal hormones in males as a group and females as a group has not revealed an effect of hormonal status. Reavis and Overman (2001) verified hormonal status (estradiol, progesterone, and testosterone) in young and older adults by blood sample assay. This resulted in six distinct groups: men: young males (mean age = 19.1) older males (mean age = 59.4). Women: young females low hormones (mean age = 19.8), young females with high hormones (mean age = 22.4), older post-menopausal females on ERT (mean age = 54.5), older post-menopausal females not on ERT (mean age = 62.7). As expected, males were significantly higher (by a factor of 20) in levels of testosterone than females. Results across 150 real/virtual card IGT trials revealed no significant differences in IGT performance (advantageous Decks C + D) between young and old groups or between the two male hormone groups or among the four female hormone groups. Thus, variations in menstrual cycle do not affect IGT performance in terms of selection of advantageous cards.

**FIGURE 6:**
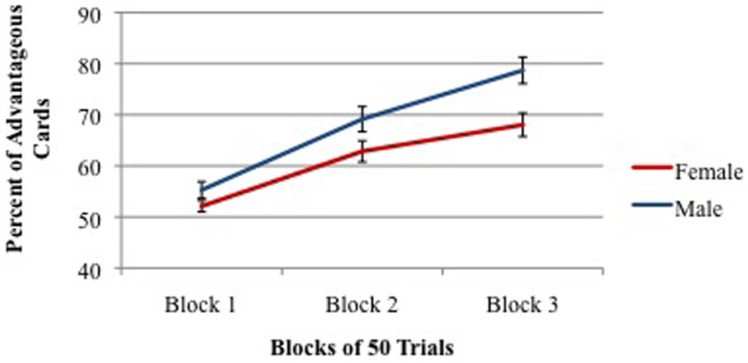
**Percent of advantageous card selections across three blocks of 50 trials for males (young + old) and females (young + old).** Vertical bars indicate SEM.

However, there was a significant gender difference with males choosing 67.7% advantageous cards and females choosing 60.7% advantageous cards. As shown in **Figure 6**, males and females were equal in IGT performance in the first block of trials, but males scored significantly higher in Blocks 2 and 3. Also, significantly more males (68%) stated the correct rule of which decks are the two good decks than did females (48%). Furthermore, males stated the correct rule significantly earlier in the task (75th trial on average) than did females (97th trial on average).

Our findings have been recently replicated by van den Bos et al. (2013), who found that while both males and females learn to choose advantageous cards across the task, males choose more cards from Decks C and D and while females chose more cards from Decks B–D. This once again reveals females’ differential preference for cards from HFOG Deck B as reported above.

### TESTOSTERONE AS A POSSIBLE CONTRIBUTOR TO IGT GENDER DIFFERENCES

Hormone assays in the study by Reavis and Overman (2001) revealed that, as expected, males have significantly more testosterone than females (1.88 vs. 0.09 ng/ml blood). Furthermore, males outperformed females on the IGT. This raises the question of a link between testosterone and differential IGT performance and perhaps a link with the ORBPFC. As shown in an experimental double dissociation, perinatal testosterone in monkeys accelerates the functional maturation of ORBPFC and slows the functional maturation of area TE in the inferior temporal lobe (see Overman and Bachevalier, 2001). In brief summary, infant male monkeys significantly outperform females on an object reversal task that is dependent upon ORBPFC. Exposure to perinatal testosterone renders female monkeys equal to males on reversal learning, and lesions of ORB in adult monkeys dramatically impair reversal learning in both males and females. In the second part of the dissociation, infant female monkeys outperform males on a TE-dependent concurrent discrimination task; however, castrated infant males perform as well as normal females and better than normal males on this task (Overman and Bachevalier, 2001).

Perinatal testosterone status is very similar in infant monkeys and humans. Thus, one might predict that young male children would outperform young female children on the object reversal task. This is exactly the finding from studies in our laboratory (Overman et al., 1996a,b). Here, children were tested with non-verbal procedures exactly as in previous studies with monkeys. The findings from those studies were as follows: (1) Male and female children performed equally on object discrimination tasks, indicating no differences in general learning ability. (2) Males under the age of 34 months were superior to age-matched females in object reversal learning with a pattern of results almost exactly like that in infant monkeys. (3) In addition to slower learning relative to males, about 20% of female children under the age of 29 months showed hyperemotional behaviors commensurate with the start of reversal training. (4) Females under the age of 36 months were superior to age-matched males in learning a concurrent discrimination task.

Thus, infant male and female children display differences in their learning abilities almost exactly like those shown by infant monkeys and, in infant monkeys, task performance is clearly dependent on perinatal differences in testosterone. With important implications for IGT performance (which is dependent upon ORBPFC), one of the monkey/child tasks, object reversal, is also known to be dependent upon functions of the ORBPFC, and the functional maturation of which depends upon testosterone. All of these data are suggestive for a “testosterone/ORBPFC dynamic” that is related to gender differences on the IGT. This dynamic is undoubtedly complex, for as shown in the next section there is strong evidence that different regions of ORBPFC are related to performance on the IGT in males and females. However, participation of the ORBPFC in the IGT is clearly not the whole story as other regions of the PFC, such as the DLPFC, have been implicated in IGT performance (e.g., Bechara, 2007).

### IMPLICATIONS FOR INVOLVEMENT OF ORBPFC: WIN–LOSS SENSITIVITY AND RISK TAKING

In a comprehensive review of sex differences on the IGT, van den Bos et al. (2013) evaluated performance results from six risk-taking tasks in order to explore sensitivity to punishment as a possible explanation for IGT gender differences. These authors concluded that performance data from these six tasks, as a group, do not support win–loss sensitivity as an explanation for IGT gender differences. However, when analyzing results specifically from the Cambridge Gambling Task (Deakin et al., 2004; van den Bos et al., 2012) and the Risky Gain Task (Lee et al., 2009) van den Bos et al. (2013) propose that *oversensitivity to loss* may be driving female performance in risk-taking tasks.

In contrast to the hypotheses of van den Bos et al. (2013), others have interpreted the IGT gender difference as being driven by a male aversion to loss and a female preference for reward (Overman et al., 2006). This interpretation is based upon the results of an PET imaging study by Bolla et al. (2004) who reported that while performing the IGT, males and females showed differential activation in subregions of ORBPFC. Specifically, males showed increases in activation in large regions of lateral ORBPFC (BA 47), while females showed increases in activation in regions of medial ORBPFC (BA 11). This is important because O’Doherty et al. (2001) have shown that in humans, the lateral ORBPFC is sensitive to punishment, whereas the medial ORBPFC is involved in reward and guessing when outcomes are uncertain. With these differences in mind, Overman et al. (2011) attempted to disrupt or override lateral OFC activity in men and render them more similar to females in IGT performance. These authors presented different aromas to participants every 10 trials during a 200 trial real/virtual card IGT, with the hypothesis that since medial ORBPFC was shown by Bolla et al. (2004) to increase in activation in females during IGT, that presentation of aromas might increase activation in males and females and render the genders equivalent in IGT performance. Female IGT performance was predicted to remain at the normal low level because of a floor effect. Indeed, when aromas were presented (and medial ORB putatively increased in activity), male IGT performance declined to the level of females. Furthermore, males who received aromas performed significantly below that of control males who did not experience aromas. If it were true that increasing activation in medial ORBPFC resulted in males adopting a reinforcement strategy more like that of females, then they (males) they might show a preference for the HFOG cards of Deck B. That is exactly what the data confirmed. In the control no-aroma IGT task, females chose significantly more cards from Deck B than did males, but in the aroma IGT task, males chose a number of cards from Deck B equal to that of females.

These results support the hypothesis that IGT sex differences may be driven, in part, by differential pattern of activation in brain regions, particularly subregions of the ORBPFC, which, in turn leads to differential sensitivity to reward and punishment by males and females.

## DISCUSSION

### OVERVIEW

There are five main points in this review, each of which is discussed below.

(1) In the past 20 years, there have been many different procedural task variations in IGT research, making it difficult to directly compare results across studies. If one is to make accurate comparisons of IGT performance across various populations, it is necessary to have a baseline of optimal performance among normal participants. Two variables result in optimal performance in non-clinical, college-age participants: the use of a combination of real and virtual cards and the administration of more than 100 IGT trials. Optimal performance on the PAR^™^ IGT does not occur with the use of virtual cards alone across 100 trials.

(2) The use of real/virtual card IGT procedures has revealed a positive linear relationship between IGT performance from ages 11 to 25 years. We did not find a significant difference in performance between normal young adults and older adults up to 65 years of age. However, there have been reports that subgroups of older adults may be impaired on the IGT.

(3) The use of real/virtual card IGT procedures has revealed a significant gender difference in performance with males choosing more advantageous cards than females. This difference exists from adolescence to old age.

(4) A deck-by-deck analysis reveals that the gender difference is driven by females’ preference for cards from HFOG decks, B and D. The gender differences are not due to differences in math ability, response perseveration, or hormonal differences within gender.

(5) The IGT gender difference may be related to a dynamic between testosterone and orbital prefrontal systems.

### ENHANCED IGT PERFORMANCE WITH REAL/VIRTUAL CARDS

Our comparison of performance on five versions of the IGT, including four versions of the PAR^™^ IGT, demonstrated that the use of virtual cards alone did not result in optimal performance in non-clinical, college-age participants. IGT procedures that employed both real and virtual cards yielded significantly higher scores in two measures of performance. First, as shown in **Figure 1**, choice of advantageous cards across 100 trials was significantly higher with the use of real/virtual cards. Secondly, as shown in **Figure 2**, the difference was more pronounced when examining performance in blocks of the task, especially in later blocks. With both procedures, significant learning (choice of advantageous cards) occurred by the second block of trials, as shown in previous reports (e.g., Bechara et al., 2000; Overman et al., 2013), but as also shown in both of these reports, the use of virtual cards alone appears to produce little if any additional learning beyond the second block. Furthermore, our data clearly show that participants achieve significantly higher performance when they are administered 200 IGT trials.

#### Implications for marker hypothesis (SMH)

These findings have two very important implications for the SMH and for the use of the PAR^™^ IGT. In IGT tests of the SMH (Damasio et al., 1991), Bechara et al. (1997) propose that the level of cognitive understanding continues throughout the IGT. Specifically, conscious realization that Decks C and D are advantageous is said to arise after approximately the 80th trial (Bechara et al., 1997, but see Maia and McClelland, 2004, who argue that participants may have knowledge about the decks early in the game). It would seem that selection of advantageous cards would increase with awareness that they are “good.” This appeared to be the case when real cards were used (Bechara et al., 1994). In these data, the single “typical control” showed increasing selection of advantageous cards throughout the task: choosing 56, 72, 80, and 96% respectively in the four blocks of 25 trials. In our study, increasing performance across the task was seen only when real/virtual cards were used but not with virtual cards only as in the PAR^™^ IGT. This brings up the question of whether all components of the SMH (i.e., emotional and conscious) come into play in the computerized IGT.

#### Implications for the PAR^™^ IGT

Taken together, our data and the data from Bechara result in a conundrum. First, the SMH (Damasio et al., 1991) was tested and supported with IGT procedures using real cards (Bechara et al., 1994, 1997). Secondly, our data and (perhaps) those by Bechara et al. (2000) show that selection of advantageous cards increases throughout the task with the use of real cards but not with the use of virtual cards. However, the PAR^™^ IGT utilizes only virtual cards. Thus, when using the PAR^™^ IGT, accurate characterization of clinical and non-clinical populations becomes problematic.

### PERFORMANCE ACROSS AGE

It is clear that performance on the IGT or similar child-friendly tasks improves from approximately ages 6–25 years. During adolescence and early adulthood (approximately 11–25 years), IGT performance improves significantly (Hooper et al., 2004; Overman et al., 2004). With regard to adolescents’ “real world” decision-making, the incidence of risky decisions decreases during the same time period in which performance on the IGT increases (see Spear, 2000). This is a period of significant changes in brain connectivity that occur throughout the brain, including in the frontal lobe and the PFC (which is closely involved in decision-making in general and the IGT in particular). Since the ORBPFC is strongly involved with IGT performance, it is an easy speculation to suppose that improved IGT performance has its underpinnings in the functional maturation of the ORBPFC and related networks. Of course, there are numerous social and environmental changes that concomitantly occur with IGT improvement during this time frame. Undoubtedly, there are complex interactions between changing brain systems and changing external variables.

It is less clear whether there are significant changes in IGT performance between young and elderly non-clinical adults. Several laboratories have failed to find significant changes between younger and older adults using virtual card IGT (Wood et al., 2005) or real/virtual card IGT (Reavis and Overman, 2001), see **Figure 5** in this review. Of particular note is the fact that the normative data used to validate the PAR^™^ IGT reveal little, if any, change in IGT performance from ages 18 to 79 years (Bechara, 2007). These data report advantageous card selection in three age groups of normal adults: young (18–39 years), older (40–59 years) and elderly (60–79 years), in which on average the scores were 60.5, 58.5, and 57%, respectively. While no statistical comparisons are presented in the PAR^™^ IGT manual, there is almost certainly not a significant difference between these three groups given the normal variance that occurs during IGT performance. However, by using subgroup analyses of IGT performance, others have collected data that suggest there are more “impaired” older adults (56–85 years) as compared to younger adults (18–55 years; Denburg et al., 2006; Fein et al., 2007).

### DIFFERENTIAL PERFORMANCE BY MALES AND FEMALES

#### Basic findings that males choose more advantageous cards

The finding that males, as a group, choose significantly more advantageous cards than females is a robust finding across several IGT procedures (see van den Bos et al., 2013). In terms of the larger IGT literature, three points are of particular importance. (1) Most IGT studies have not analyzed for gender. (2) Gender differences may not be apparent in 100 IGT trials; however, they become apparent when performance is analyzed across 200 trials, and in particular, in the latter blocks of trials. (3) A deck-by-deck analysis reveals more detailed information about the behavioral underpinnings of the gender difference. Specifically, females perform lower than males because they have a tendency to select more cards from Deck B, which is a HFOG deck. It would seem to follow that to best understand IGT performance in clinical populations, studies should include gender analyses, more than 100 trials, and deck-by-deck analyses.

#### How to report IGT data

The procedure of a deck-by-deck analysis raises the question of how best to report IGT performance. Net score reports (advantageous cards minus disadvantageous cards) cannot reveal information about preferences for different decks. Information about deck selection is better presented in terms of percentage scores. Furthermore, net score data are actually transformations of more readily understandable data using percentages. For example, a comparison of net scores of 6 vs. 10 is not easily interpreted as 65 vs. 75% advantageous cards were selected. The transformation from percent to net score does not seem to be necessary, and in fact, it prevents a complete analysis of performance.

#### Deck-by-deck analysis and gender differences

The value of a deck-by-deck analysis is clearly shown in a recent review of IGT studies (Steingroever et al., 2013). These authors argue that individual deck analysis reveals critical information about the process of decision-making during the IGT. For example, in a review of 17 studies, Steingroever et al. (2013) show that cards from Deck B (disadvantageous with infrequent losses) are chosen as often as the two good decks (C and D). The implication is that subjects are choosing on two factors (a) preference for advantageous decks that yield long-term gain and (b) preference for cards with a HFOG, even though they may be disadvantageous in the long run. Our results add another twist to this analysis: females drive the preference for high-frequency-of-gain cards. There are additional studies that report “high frequency of gain” preference (Chiu and Lin, 2007; Lin et al., 2007; Chiu et al., 2008). However, the data in these papers are difficult to interpret for two reasons. First, none of the reports analyzed/reported effects of gender. Secondly, these authors employ task versions that differ significantly from the mainstream IGT procedures. For example, in the IGT modification used by Chiu et al. (2008), the schedule of wins and losses repeats every five trials for each deck. This would seem to render the task much more transparent than the original IGT (e.g., Bechara et al., 1994; Overman et al., 2006).

Our results clearly show that in normal college-age participants, the lower IGT score by females is driven by their tendency to choose HFOG cards from Deck B. But females also learn to choose good decks across the task. Thus, their decision-making processes seem to be driven by frequency of gain as well as long-term gain.

#### Interpretation of gender differences

The meaning of gender differences in IGT performance is not completely understood at this time. The differences are not due to gender differences in math ability, response perseveration, or hormone fluctuations within females (Reavis and Overman, 2001; Overman et al., 2004). There are some interesting speculations relating differential IGT performance by males and females to differences in females’ preference for reward and males’ aversion to loss (Bolla et al., 2004), which, in turn, may be related to sex differences in a testosterone/ORBPFC dynamic (Overman et al., 1996b, 2011; van den Bos et al., 2013). The fact that women, as a group, do not perform as highly as men on the IGT should not be interpreted to mean that females are “inferior decision makers.” Such speculation does not agree with fact that significantly more males than females make poor “real-life” decisions, e.g., regarding substance abuse and gambling. Rather, it appears that within the context of the IGT, some, but not all, females are responding differently than males to specific components of the task.

## SUMMARY

The IGT has been an incredibly fruitful research tool during the past 20 years. However, the findings from the multitude of the studies are difficult to integrate and interpret due to wide variations in task methodologies and analyses. Dunn et al. (2006) make a valuable effort to integrate data from a variety of IGT studies in order to critically evaluate the SMH. However, they suggest that there are a variety of potential designs that could be used to better elucidate understanding of IGT decision-making as well as the SMH. In this review, we argue that a more complete understanding of IGT phenomena will best evolve if the performance of all populations are compared and contrasted to a common baseline of optimal performance. We believe that this baseline is established by the use of real/virtual cards across more than 100 trials, deck-by-deck analyses, and analyses for gender.

## Conflict of Interest Statement

The authors declare that the research was conducted in the absence of any commercial or financial relationships that could be construed as a potential conflict of interest.
